# Divide and Conquer: Enriching Environmental Sequencing Data

**DOI:** 10.1371/journal.pone.0000830

**Published:** 2007-09-05

**Authors:** Anne Bergeron, Mahdi Belcaid, Grieg F. Steward, Guylaine Poisson

**Affiliations:** 1 Computer Science, Université du Québec à Montréal, Montreal, Canada; 2 Information and Computer Sciences, University of Hawaii at Manoa, Honolulu, Hawaii, United States of America; 3 Department of Oceanography, University of Hawaii at Manoa, Honolulu, Hawaii, United States of America; University of Liverpool, United Kingdom

## Abstract

**Background:**

In environmental sequencing projects, a mix of DNA from a whole microbial community is fragmented and sequenced, with one of the possible goals being to reconstruct partial or complete genomes of members of the community. In communities with high diversity of species, a significant proportion of the sequences do not overlap any other fragment in the sample. This problem will arise not only in situations with a relatively even distribution of many species, but also when the community in a particular environment is routinely dominated by the same few species. In the former case, no genomes may be assembled at all, while in the latter case a few dominant species in an environment will always be sequenced at high coverage to the detriment of coverage of the greater number of sparse species.

**Methods and Results:**

Here we show that, with the same global sequencing effort, separating the species into two or more sub-communities prior to sequencing can yield a much higher proportion of sequences that can be assembled. We first use the Lander-Waterman model to show that, if the expected percentage of singleton sequences is higher than 25%, then, under the uniform distribution hypothesis, splitting the community is always a wise choice. We then construct simulated microbial communities to show that the results hold for highly non-uniform distributions. We also show that, for the distributions considered in the experiments, it is possible to estimate quite accurately the relative diversity of the two sub-communities.

**Conclusion:**

Given the fact that several methods exist to split microbial communities based on physical properties such as size, density, surface biochemistry, or optical properties, we strongly suggest that groups involved in environmental sequencing, and expecting high diversity, consider splitting their communities in order to maximize the information content of their sequencing effort.

## Introduction

Whole genome shotgun sequencing is a standard approach for quickly achieving a high degree of genome coverage for individual organisms. This procedure is now also being applied to environmental sequencing projects in an approach commonly referred to as metagenomics or microbial community genomics [1]. For this application, a community shotgun library is prepared from DNA that has been extracted from a natural assemblage of microorganisms, rather than from an individual isolate. Creation of such a library is a convenient way to capture the full spectrum of microbial genetic diversity within a particular sample, but the library is a jumble of genome fragments from many different microbial species or strains, often numbering in the thousands to hundreds of thousands or more [1], [2]. As a consequence, random sequencing of clones from a metagenomic library often results in a low proportion of overlapping fragments. Much of the power of genomics derives from understanding genes in their genomic context, and the failure to assemble individual sequence reads (singletons) into longer stretches (contigs) represents a significant loss of genomic information that was originally present in the sample.

It has been proposed that physical fractionation of a microbial community prior to metagenomic analysis should improve the assembly process by reducing the complexity within each of the resulting fractions [3], but neither the specific conditions under which this should be true nor the magnitude of the benefit have been critically examined. The benefits of fractionation are most obvious in cases where a single population of interest is selectively enriched from a more complex community. In this case, all of the sequencing effort can be focused on a single fraction that is highly enriched in the population of interest, and genome reassembly for that population is improved [4]. What is unclear is whether, and to what degree, fractionation improves assembly in a more general sense, i.e., in cases where a single population is not specifically targeted and sequencing effort is distributed evenly among fractions.

In preparation for an investigation of marine viral diversity, we wished to quantify the possible benefits of splitting a complex viral community into fractions prior to library construction. Viruses make a particularly appropriate case study for examining this question. In practice, metagenomic analyses of viral assemblages have yielded very low frequencies of contigs [5], [6], [7], so any steps that could be taken to improve assembly would be useful. At the same time, viruses are amenable to physical fractionation by a variety of centrifugation [8], [9] and chromatographic [10], [11], [12] techniques, which means that if benefits of fractionation can be established theoretically this knowledge might be readily translated into practice.

To test the theoretical benefits of fractionation, metagenomic library construction and analysis were modeled for virtual viral communities, with known structure and diversity, that were either kept intact or split into fractions having non-overlapping sets of populations. Total sequencing effort was the same in all cases, but was divided evenly between fractions in the case of split communities. The proportion of sequences contributing uniquely to a contig was used as an index of assembly success. The model provides a theoretical basis for understanding the effects of fractionation on sequence assembly and predicts the specific conditions under which fractionation should be beneficial.

## Results and Discussion

In the first section we present a simple model that assumes that species are uniformly distributed in a community. While this assumption might be far from the biological reality, its mathematical tractability allows us to discuss, in a simple setting, results that upgrade to more complex models presented in the subsequent section.

### The Uniform Distribution Model

In this first model, we work with a community of *M* different species, with genome length *G* in base pairs (bp), and we assume that all the species have the same abundance. An environmental sequencing project is described by the following parameters:

1. *N* : the number of fragments that are sequenced,
2. *L* : the average length in bp of each sequence,
3. *T* : the minimum overlap, in bp, required to assemble sequences.

Throughout this paper, we will consider *N*, the *sequencing effort*, as a constant. The value of *L* depends on the sequencing quality and approach, Sanger-like or pyrosequencing [13], and the value of *T* is used as a threshold in the assembly process.

Given this model, it is possible to evaluate the expected number of singletons that are sequences that do not overlap any other, in the assembly. Using the Lander-Waterman model [14], [15], we have:

#### Claim 1

Under the uniform distribution assumption, the expected number of singletons of an environmental sequencing project is:
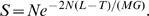



When a community is split into two sub-communities, the number of species in the two sub-communities can be represented by *pM* and *(1−p)M*, where *p* is a number between 0 and 1. We are interested in comparing the effect of splitting the original *N*-sequences project into two *N/2*-sequences projects, one for each of the two sub-communities. Many different measures can be used to compare assemblies, and we begin with a very simple measure: we compare the number of singletons in each assembly. In metagenomics projects, singletons typically form a huge proportion, often more than half, of the sequencing effort. On the other hand, large contigs are a rarity, making the usual measures of assembly quality almost useless.

Let *S*_1_+*S*_2_ be the sum of the expected numbers of singletons resulting from the two *N/2*-sequences projects. If *S>S*_1_+*S*_2_, then the split project has assembled more sequences than the original project. We will refer to the difference *S−*(*S*_1_+*S*_2_) as the number of *assembled singletons* resulting from the split. We have:

#### Claim 2

Under the uniform distribution assumption, if
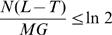
then *S−*(*S*_1_+*S*_2_)≥0, for all possible values of *p*. Furthermore, *S−*(*S*_1_+*S*_2_) = 0 when *p* = 0.5.

The significance of Claim 2 is better explained by computing the quantity *N*(*L*−*T*)/(*MG*) with realistic values. The following sequencing project of a community of phages is inspired by the parameters and diversity estimates of [7]:

1. *M* : 5000 species of phages,
2. *G* : average genome length of 50 000 bp,
3. *N* : 400 000 fragments sequenced,
4. *L* : average sequence length of 102 bp,
5. *T* : minimum overlap of 35.

**Figure 1:**
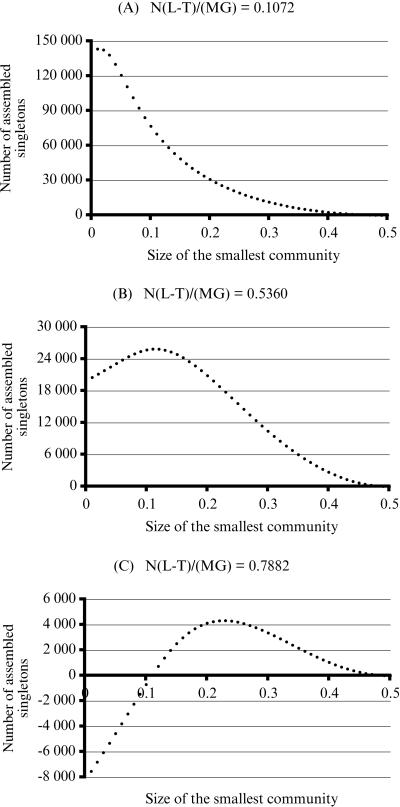
These three curves depict the gain (loss) in assembled singletons when a 400 000 sequences project is divided equally into two 200 000 sequences projects on sub-communities of increasing sizes, assuming uniform abundance. The values of the horizontal axis are the sizes, in fraction, of the smaller sub-communities. In curve (A), the total number of species is 5000, thus *N*(*L*−*T*)/(*MG*) = 0.1072 In curve (B), the total number of species is 1000, and *N*(*L*−*T*)/(*MG*) = 0.5360 approaches ln 2. In curve (C), with only 680 species, *N*(*L*−*T*)/(*MG*) = 0.7882 exceeds ln 2, and losses are observed when the smallest subcommunity is too small.

With these values, *N*(*L*−*T*)/(*MG*) = 0.1072, which is indeed smaller than ln 2 = 0.6931… For *p* = 0.1, the values of *S* and *S*_1_+*S*_2_ are respectively 322 810 and 246 007 which means that, when the community is split into two sub-communities containing respectively 10% and 90% of the original species, the same sequencing effort will yield 76 804 more assembled singletons. Figure 1(A) shows the gain in overlapping sequences for this experiment, for values of *p* between 0.01 and 0.5. It is interesting to note that splitting the species into two almost equal sub-communities is both highly unlikely from a biological point of view, and undesirable from a computational point of view.

The value *N*(*L*−*T*)/(*MG*) augments proportionally to the *coverage*, defined as *NL*/(*MG*), which is the expected number of times a single base pair will be sequenced. When coverage augments, the benefits of splitting the community gradually disappear. If we lower the diversity of the preceding experiment to *M* = 1000, then the value of *N*(*L*−*T*)/(*MG*) is still smaller but close to ln 2, and the gain in assembled singletons is more modest, as can be seen in Figure 1(B).

Finally, when *N*(*L*−*T*)/(*MG*) becomes greater than ln 2, with a diversity of *M* = 680 for example, then losses of assembled singletons occur when the smallest community is too small. Figure 1(C) shows that these losses occur when the smallest sub-community represents less than 10% of the population. This phenomenon is explained by the fact that, at low coverage, the number of singletons grows with the sequencing effort, but as coverage augments, the number of singletons peaks, and eventually shrinks when substantial parts of the genome are assembled. A loss of assembled singletons is not necessarily bad, since many of the species of the small sub-community are sequenced at a high coverage (more than 6× for *p* = 0.1). This could produce a high number of complete genomes of sparse species, showing that merely counting the number of singletons in an assembly is a very crude way to compare assemblies.

When *N*(*L*−*T*)/(*MG*) = ln 2, then, by Claim 1,
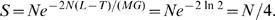



This means that, under the uniform distribution assumption, if a sequencing project is expected to produce at least N/4 unassembled sequences, or 25% of the sequencing effort, then splitting the community is always a wise strategy.

This apparently counter-intuitive result can be explained by the following observations. For highly diverse communities, or for large genomes, the number of singletons initially grows linearly with the sequencing effort: doubling the sequencing effort doubles the number of singletons. However, this is not true for less diverse communities, or for smaller genomes. Consider, for example, a large jigsaw puzzle. If a group of pieces is picked at random, most of them will not fit together, even if the number of selected pieces is doubled. On the other hand, the reverse effect is observed as the number of pieces increases with respect to the size of the puzzle. Selected pieces that do not fit together are less frequent, and eventually vanish.

By combining these two behaviors, we explore the window in which fractionation yields both a better assembly, for the small sub-community, and a reasonable sampling of the diversity of the original community. The jigsaw puzzle analog of splitting a community would be the fairly common strategy of sorting out pieces of a given color, in the hope of assembling in parallel a smaller but significant part of the big picture. This strategy works best when the selected color does not cover half of the area (blue sky with small patches of clouds) or only a tiny rectangle (a little red house in the mountain). Physical separation of species, like sorting puzzle pieces by color, requires knowledge and yields information. It is this information that is used to get better assembly results with the same sequencing effort. Of course, the cost of getting this information must also be considered when planning a project.

### Non-uniform Models

When the species of a community are not uniformly distributed, the mathematical analysis of the effects of splitting a community is much harder, and always depends on the exact distribution. Since the structure and diversity of actual microbes community is still largely unknown, we choose to attack the problem using simulations with a distribution of *M* = 4991 surnames found in a fixed geographical location, the Province of Quebec, that had a long tradition of giving the surname of the father to his children. Each surname is identified to a species. A more detailed presentation of this community, called Quebec-Ohana, can be found in the Material and Methods section. A second community, Quebec-Ohana-Truncated, is formed by the 1319 most abundant species of Quebec-Ohana.

Again, let *N* be the number of fragments sequenced. A simulated environmental sequencing project draws a sample of *N* individuals in the community, with a probability for an individual to be selected proportional to the abundance of its species. Then, given the number of times a species is sampled, it is possible to compute the expected number of singletons contributed by each species in the sample (see Material and Methods). Splitting a community into sub-communities was done by random choices, and all the results were averaged on 10 different splits, for each value of *p*. We used the values of the last section for parameters *G*, *N*, *L* and *T*.

Finally, in order to be able to compare similar experiments, we performed simulated environmental sequencing on a uniformly distributed community of 4991 species. Table 1 gives detailed statistics of simulated assemblies when these three communities are split into two sub-communities containing respectively 10% and 90% of the original species.

**Table 1:** Statistics on the number of singletons, before (*S*) and after (*S*_1_+*S*_2_) a split 10%–90%, for a total sequencing effort of 400 000.

Community	*S*	Percentage of singletons	*S*_1_+*S*_2_	Percentage of singletons	Gain in assembled singletons	Percentage of gain
Uniform	321915	80.5	245288	61.3	76626	23.8
Quebec-Ohana	169316	42.3	141983	35.5	27333	16.1
Quebec-Ohana	121661	30.4	98757	24.7	22903	18.8
Truncated						

The biggest gain in assembled singletons is observed in the uniformly distributed community, and is still important in the two other communities. The gain for the uniform distribution, 76 626, is highly consistent with the predicted result of last section (the model gave an expected gain of assembled singletons of 76 804 for *M* = 5000). This distribution also has the highest percentage of singletons, 80.5%, in the 400 000-sequences project. The two other distributions have a lower percentage of singletons in the 400 000-sequences project, respectively 42.3% and 30.4%, but the percentages of the number of assembled singletons over the number of original singletons is comparable for all three distributions, ranging from 16.1% to 23.8%. It is interesting to note that recent environmental sequencing projects [16] have percentages of singletons (53%) that are comparable to the percentages that we obtained in these three experiments.

**Figure 2:**
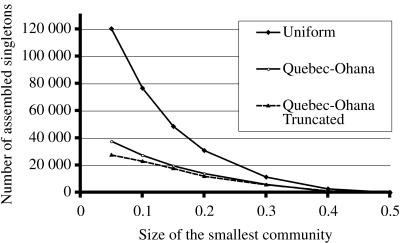
These three curves show the gain in assembled singletons when a 400 000 sequences project is divided equally into two 200 000 sequences projects on sub-communities of increasing sizes, for three communities with different structure and diversity. The values of the horizontal axis are the sizes, in fraction, of the smaller sub-communities. For the top curve, the community has 4991 equally abundant species. For the middle curve, the community has 4991 species whose abundance distribution mimics the distribution of surnames in the Province of Quebec. The community for the bottom curve is formed by the 1319 most abundant species of the preceding community.

Figure 2 shows the gain in assembled singletons, for values of *p* between 0.05 and 0.5. The trends observed in the theoretical results on uniform models are clearly visible. All three curves show that the greatest advantages are obtained when the two sub-community are split unequally, and the comparison between the two Quebec-Ohana communities shows that the higher the diversity, the higher the benefits of splitting.

### Relative Diversity

In this section, we investigate the possibility of recovering the relative diversity of two sub-communities resulting from a split, given their comparative assembly statistics.

**Figure 3:**
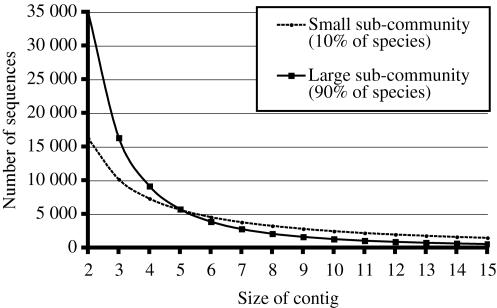
This figure shows parts of the spectra of assemblies resulting from a split of a 400 000 sequences simulated project of Quebec-Ohana in two sub-communities representing, respectively, 10% and 90% of the species. The small sub-community has fewer small contigs than the large sub-community, but more larger contigs, for sizes greater than 5.

In the simulations, apart from the number of singletons, we also computed the expected number of sequences that participate in contigs of size 2 to 100 (see Data S1). These series of values will be called the *assembly spectrum*. When a community of *M* species is split into two sub-communities of *pM* and (1−*p*)*M* species, it is thus possible to compare not only the number of singletons, but also their whole spectra. Figure 3 gives an example of two (partial) spectra for a split of Quebec-Ohana in sub-communities representing 10% and 90% of the species, and for contig sizes from 2 to 15.

**Figure 4:**
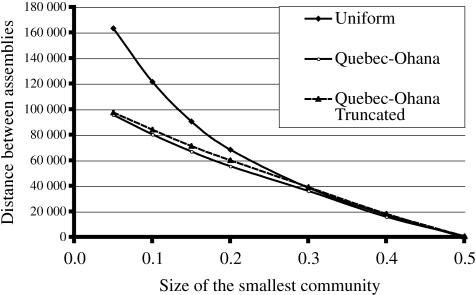
These three curves show the distances between the two assembly spectra obtained by splitting equally a sequencing effort on two sub-communities of *pM* and (1−*p*)*M* species, for values of *p* from 0.05 to 0.5, and for three communities with different structure and diversity. For all values of *p*, the two curves corresponding to Quebec-Ohana communities are very close. For *p* larger than 0.3, the three curves are almost identical.

In order to compare spectra, we computed the Euclidian distance between the two spectra, that is, if *a_q_* and *b_q_* represent, respectively, the number of sequences that participate in contigs of size *q* in each assembly, we computed:
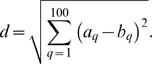



These values are shown in Figure 4, for values of *p* between 0.05 and 0.5, for all three communities.

The surprising finding of this experiment is that, when the small sub-community represents more than 30% of the entire community, the behavior of the distance is almost the same for the three communities. This implies that, if the distance between two assemblies is lower than 40 000, then the relative diversity of the two sub-communities can be recovered, independently of the abundance distribution of the original population. For example, a distance *d* = 20 000 would imply that the entire community was split in sub-communities containing approximately 40% and 60% of the original species.

For values of *p* between 0.05 and 0.3, the distance curves for Quebec-Ohana and Quebec-Ohana-Truncated are also very similar, despite the fact that these two communities have different structure and diversity. For low values of *p*, the community with uniform abundance has a diverging behavior, with much greater distances between assemblies, suggesting that distances over 120 000 could indicate that the community has indeed a uniform abundance distribution.

### Conclusions

Our results imply that when diversity is high, as in most natural viral communities [2], pre-fractionation of a community almost always improves the overall proportion of assembled sequences. An implied corollary of this result is that pooling of samples [7], is likely to lead to a loss of information compared to that which would be obtained by evenly dividing the same sequencing effort among libraries prepared from the individual samples.

Gaining some practical benefit from this theoretical insight could involve relatively minor adjustments to current protocols for viral metagenomics and relatively little extra effort. Viral assemblages are usually purified by banding in density gradients prior to metagenomic library construction [2]. Since this procedure also separates populations of viruses based on differences in their buoyant density [17], viruses could simply be harvested from a density gradient as two or more density fractions. Metagenomic libraries from the two fractions could then be constructed and sequenced as separate samples. Even making this simple adjustment in strategy has the potential to increase the frequency of contigs per unit sequencing effort.

## Material and Methods

### The Uniform Distribution Model

In this section, we give formal proofs of Claim 1 and Claim 2. We first recall the relevant results of the Lander-Waterman model [15], which is adapted to traditional sequencing projects of the genome of one species. Define:

1. *G* : the length of the genome,
2. *N* : the number of fragments that are sequenced,
3. *L* : the average length in bp of each sequence,
4. *T* : the minimum overlap, in bp, required to assemble sequences.

Then the expected number *S* of singletons in the assembly is
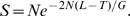

*Proof of Claim 1:* Suppose a community of *M* species has uniform abundance distribution, with all species having the same genome size *G*. If a total sequencing effort of *N* sequences is applied to this community, we can expect that each species will contribute *N*/*M* sequences to the project. Applying the Lander-Waterman model to each species yield the following expected number *S′* of singletons from each species:
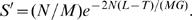
Since there are *M* species, the total expected number of singletons will be
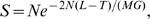
and this completes the proof of Claim 1.

The proof of Claim 2 relies on the following two lemmas whose – rather technical – proofs are available in Proofs S1.

#### Lemma 1

If *x*>0 then 
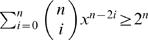


#### Lemma 2

If *x*>0 and α>0 then *a^x^a* ^1/*x*^−(*a^x^*+*a* ^1/*x*^)≥*a*(*a*−2).

*Proof of Claim 2:* If the community of *M* species is split into two sub-communities of *pM* and (1−*p*)*M* species, and if the sequencing effort is distributed equally between the sub-communities, then the expected number of singletons *S*_1_+*S*_2_ from the two projects will be:
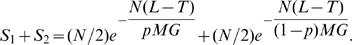



Let *a* = *e^N^* ^(*L−T*)/(*MG*)^ and *x* = (1−*p*)*/p*. We will prove that *x*>0 and α≤2 implies *S−*(*S*_1_+*S*_2_)≥0. Using α and *x* yields the following expressions for the gain in assembled singletons:
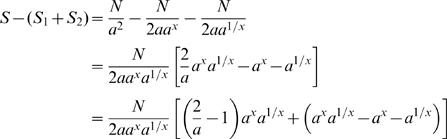



Since α≤2, the first term of the sum is positive. By Lemma 1, *x*+1/*x*≥2, implying that *a^x^a* ^1/*x*^≥*a* ^2^. Applying this bound and Lemma 2 to the second term yields the following:
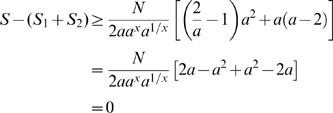
This completes the proof of Claim 2.

### Quebec-Ohana: A Community of Surnames

The Province of Quebec has a unique history in North America. Starting in 1608, a few thousand French settlers occupied the territory, which then passed, in 1760, under British rule. The decision of the British administration to allow the French settlers to keep their language and religion resulted in the effective isolation of this community. The specific characteristics of the Quebec population have already been used in genetic studies (see, for example [18]). Up to 1981, the tradition to give the surname of the father to its children resulted in a current population of surnames whose distribution pattern could be similar to bacteria and viruses populations. Currently, 4991 different surnames occur with a frequency higher than 0.001% in the estimated 7.5 million residents of the province.

The “Institut de la statistique du Québec” has published a detailed distribution of the abundance of these 4991 surnames [19]. We used this distribution as a basis for constructing our test community, Quebec-Ohana, identifying each different surname as a *species* (part of Hawaiian culture, ‘ohana’ means ‘family’ in an extended sense of the term including both blood-related or extended). The abundance of each species in this community is the relative abundance of a surname in the community of 4991 surnames. The most abundant species, *Tremblay*, forms 1.29% of the population, and 16 species, all of French origin, account for 10% of the population.

At the other end of the abundance curve, 3672 species (73.6% of the species) each form 0.01% or less of the population of Quebec. Removing these species yielded a community of the 1319 most abundant species of Quebec-Ohana, Quebec-Ohana-Truncated. The abundance of each species in this community is the relative abundance of a surname in the community of 1319 surnames. The abundance distributions of these two communities are available in Data S2.

### Simulated Environmental Sequencing

In order to simulate an *N*-sequences sequencing project, we begin by sampling *N* individuals, with a probability for an individual to be selected proportional to the abundance of its species in the community. Each individual in the sample will contribute one fragment to be sequenced. From this sample, we compute:

1. *F_i_* : the number of species for which *i* fragments have been sequenced.

The next step is to compute *C_q_*, the expected number of sequences in contigs of size *q*. For a contig of size *q*, one needs *q−1* overlaps and two non-overlap gaps. The Lander-Waterman model [15] gives the probability that a randomly selected fragment is part of a contig of size *q* as:
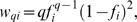
where
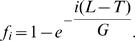
In environmental sequencing projects, there is a strong possibility that many species will contribute just a few fragments to the total project. It is thus necessary to modify the above model and add the necessary condition that a species must contribute at least *q* fragments in order to have a chance to contribute contigs of size *q* to the assembly. By adding this condition, we slightly depart from the model derived in [5].

For a species for which *i* fragments have been sequenced, the probability *p_qi_* that a randomly selected fragment is part of a contig of size *q* is thus given by:
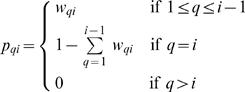



Finally, the expected number *C_q_* of sequences in contigs of size *q* is obtained by summing all the contributions of individual species:
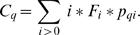



## Supporting Information

Data S1 Results of the simulations
(0.04 MB XLS)

Data S2 Structure and diversity of Quebec-Ohana
(0.96 MB XLS)

Proofs S1 Proofs of Lemma 1 and Lemma 2
(0.03 MB PDF)
