# Supplementary material for: Divide and Conquer: Enriching Environmental Sequencing Data
Source: PLoS One. 2007 Sep 5;2(9):e830. doi: 10.1371/journal.pone.0000830 (PMC1952108; doi:10.1371/journal.pone.0000830)
Supplement: Proofs S1 — Proofs of Lemma 1 and Lemma 2 (0.03 MB PDF) [file pone.0000830.s003.pdf]

Proofs

Lemma 1. If $x > 0$ then $\sum_{i=0}^{n} \binom{n}{i} x^{n-2i} \geq 2^n$.

Proof: We first show that for $k \geq 0$ and $x > 0$, $f(x) = x^k + x^{-k} \geq 2$. The case $k = 0$ is immediate. For $k > 0$, the first derivative

$$f'(x) = kx^{k-1} + (-k)x^{-k-1}$$

equals 0 if and only if $x = 1$, and the second derivative,

$$f''(x) = k(k-1)x^{k-2} + (-k)(-k-1)x^{-k-2}$$

is positive at $x = 1$.

Suppose that n is odd. We have:

$$\begin{aligned}
\sum_{i=0}^{n} \binom{n}{i} x^{n-2i} &= \sum_{i=0}^{\lfloor n/2 \rfloor} \binom{n}{i} x^{n-2i} + \sum_{i=1+\lfloor n/2 \rfloor}^{n} \binom{n}{i} x^{n-2i} \\
&= \sum_{i=0}^{\lfloor n/2 \rfloor} \binom{n}{i} x^{n-2i} + \sum_{n-i=0}^{\lfloor n/2 \rfloor} \binom{n}{n-i} x^{n-2(n-i)} \\
&= \sum_{i=0}^{\lfloor n/2 \rfloor} \binom{n}{i} (x^{n-2i} + x^{-(n-2i)}) \\
&\geq \sum_{i=0}^{\lfloor n/2 \rfloor} \binom{n}{i} 2 \\
&= 2^n.
\end{aligned}$$

When n is even, then the sum has an odd number of terms. We obtain the inequality by pairing the positive and negative powers of x as above, and by noting that the middle term of the sum:

$$\binom{n}{n/2} x^{n-2(n/2)}$$

does not depend on x.

Lemma 2. *If $x > 0$ and $a > 0$ then $a^x a^{1/x} - (a^x + a^{1/x}) \geq a(a-2)$.*

Proof: We develop the left side of the inequality in the classical power series for the exponential function:

$$a^x a^{1/x} - (a^x + a^{1/x}) = \sum_{n=0}^{\infty} \frac{(\ln a)^n}{n!} \left[\left(x + \frac{1}{x} \right)^n - \left(x^n + \frac{1}{x^n} \right) \right]$$

$$
\begin{aligned}
&= -1 + \sum_{n=2}^{\infty} \frac{(\ln a)^n}{n!} \left[\sum_{i=1}^{n-1} \binom{n}{i} x^{n-2i} \right] \\
&\geq -1 + \sum_{n=2}^{\infty} \frac{(\ln a)^n}{n!} \left(2^n - 2\right), \text{ by Lemma 1,} \\
&= -1 + \sum_{n=2}^{\infty} \frac{(2\ln a)^n}{n!} - 2\sum_{n=2}^{\infty} \frac{(\ln a)^n}{n!} \\
&= -1 + (a^2 - 2\ln a - 1) - 2(a - \ln a - 1) \\
&= a(a-2).
\end{aligned}
$$
